# Supplementary material for: The molecular architecture of the desmosomal outer dense plaque by integrative structural modeling
Source: bioRxiv. 2024 Mar 10:2023.06.13.544884. Originally published 2023 Jun 14. Preprint. [Version 2] doi: 10.1101/2023.06.13.544884 (PMC10312763; doi:10.1101/2023.06.13.544884)
Supplement: Supplement 1 [file media-1.pdf]

Supplementary Information

The molecular architecture of the desmosomal outer dense plaque by integrative structural modeling

Satwik Pasani[1], Kavya S Menon[1], Shruthi Viswanath[1,*]

[1] National Center for Biological Sciences, Tata Institute of Fundamental Research, Bengaluru 560065, India

* Corresponding Author: shruthiv@ncbs.res.in (SV)

Section 1: Modeling

Section: 1.1 Stoichiometry Runs

The number of molecules that could be accommodated in the PG layer was unclear since it was not possible to unambiguously dock the molecules manually in this region. A higher number would result in an overcrowded model or push molecules outside the tomogram space and be spatially unconstrained by the tomogram, while a smaller number would not be enough to explain the whole EM map. To determine this, we ran independent modeling runs that only included the PG and DP molecules and the PG layer EM density. We ran six simulations, each with an equal number of PG and DP ranging from 2 to 7. For each of these runs, the representation and sampling followed the IMP modeling protocol (Saltzberg et al., 2021; Viswanath et al., 2017). The restraints applied included the EM restraint, the immuno-EM restraint, the excluded volume restraint, connectivity restraint, and the cylinder restraint to ensure that the molecules are not too far away from the tomogram. 3 million models were simulated per run. For each run, after filtering the models sampled before equilibration, the top 10% models were determined and the cross-correlation coefficient of these models to the cryo-electron tomogram was computed (Bonomi et al., 2019) (Fig. S1). The number of PG/DP molecules was taken to be four. It was only slightly lower in average cross-correlation than the value for three PG/DP molecules. Selecting four PG/DP copies allowed selecting four PKPs in the PKP layer including the central PKP without introducing any asymmetries in the selection. It also allowed us to maintain 1:1:1:1 stoichiometry for PG:DP:PKP:DC with equal numbers of DSC and DSG (2 each). Finally, it was consistent with previous studies which showed that no more than four PG and DP molecules each could fit in the PG layer (Al-Amoudi et al., 2011).

Section: 1.2 Restraints

Distance threshold for sequence connectivity restraint

To set up the connectivity restraint, we need to scale the inter-bead distance to allow the more disordered N/C termini as well as the DC proteins to span a greater end-to-end distance compared to the globular protein domains. For each protein domain with at least a partial disorder (for example, PKP-N, DP-N, etc), we first find the radius of gyration of this fragment assuming the fragment to be completely disordered, using $R_g = 1.92N^{0.6}$(Kohn et al., 2004) where *N* is the number of residues. We model the fragment as a chain of monomers composed of *n* statistically independent segments each of length *a*. We assume *n* to be approximately equal to the number of beads in our representation of the fragment as two adjacent beads are free to be in any relative orientation without any other restraints; *a* is then the inter-bead distance. We then use a relation between the RMS Distance between the two ends of the chain

($R_F = an^{0.6}$) and the radius of gyration (R_G) to estimate *a* for our fragment: ${R_G}^2/{R_F}^2 = 25/176$ (Teraoka, 2002). Another estimate for *a* is calculated internally in IMP and comes from the assumption that the fragment is globular (Alber et al., 2007). The final scaling depends on the weighted sum of these two estimates, the weights corresponding to the portion of the fragment predicted to be disordered by PSIPRED (Buchan & Jones, 2019). Given an estimate of *a*, we can calculate the surface-to-surface distance for adjacent beads to create an harmonic upper bound restraint such that the beads are only penalized when they are farther than this distance apart. We use the maximum end-to-end distance (*an*) and find the bead surface-to-surface distance needed to achieve this end-to-end distance. This is approximated by the following relation where *r* is the typical radius of a bead in our model: $d = (an - 2r)/n - 2r$. The calculated scale matches the scale calculated using a more accurate measure for ${R_G}^2/{R_F}^2 \approx 0.95/6$ given by renormalization theory (Teraoka, 2002) up to rounding. However, the scale is only a heuristic parameter and the results obtained are relatively robust to its exact value.

Section 2: Analysis

Section: 2.1 Filtering based on Autocorrelation

In order to filter a computationally feasible subset of models from the large set of sampled models, we first remove the initial few models based on statistical testing (Chodera, 2016; Saltzberg et al., 2021), to consider only the models after equilibration assumed to be in the stationary distribution. Next, we only take every 20th model in the MCMC sampling run (PMI analysis parameter nskip=2, writing every 10th frame to disk). To identify an appropriate number of models to skip, we ran eight independent single-replica runs with all the restraints. We analyzed the spatial autocorrelation of the XYZ coordinates of each bead along the sampling trajectory. We chose as our cutoff the smallest number of sampling steps after which the autocorrelation of all the beads had fallen to at most 85-90%. This allows us to remove the highly correlated models to obtain an independent set of models to analyze downstream.

Section: 2.2 Cross-Correlation of Localization Densities and cryo-electron tomograms

We first computed the predicted localization density by combining the densities from all modeled proteins for the major cluster separately for the PKP layer (PKP-S) and PG-Layer (PG-N,S,C, DP-N,S). We then calculated the cross-correlation between the predicted density and the reference cryo-electron tomogram by calculating the Pearson correlation between the voxel-wise values in the two maps. This is calculated at all grid points at a voxel spacing of 5Å spread over the volume enclosing both the predicted localization density and the cryo-TM map. The values of the maps at these grid points were found by interpolation (RegularGridInterpolator in scipy (Virtanen et al., 2020)). This is similar to calculating Correlation around mean in UCSF Chimera (fitmap) except that the Chimera calculation only involves the non-zero grid points of the reference map, causing the correlation value to change depending on the order of the two maps.

Supplementary Figures

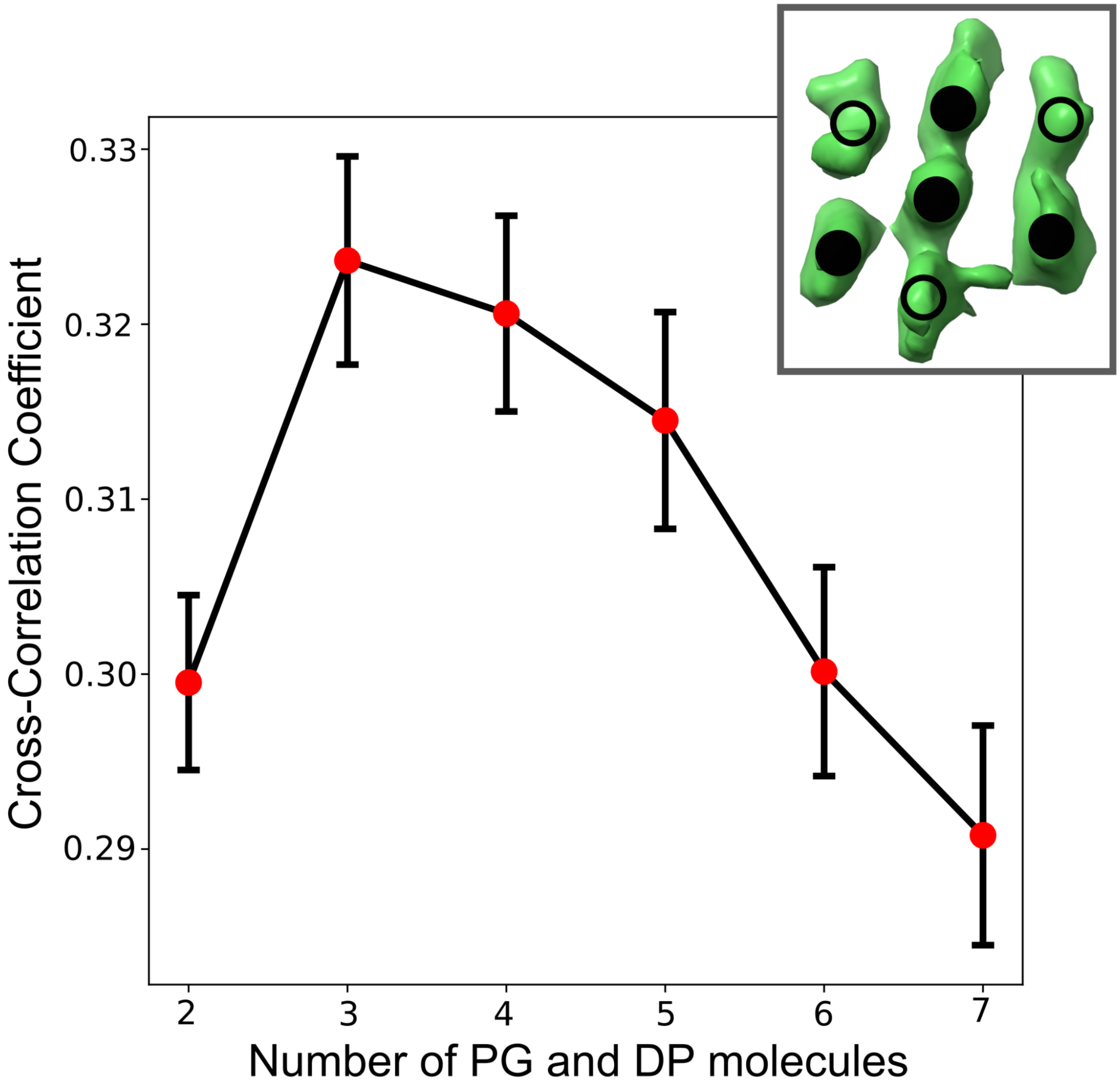

Figure S1 Estimating the number of PG and DP copies and selecting the layout for PKP copies The graph shows the results for independent stoichiometry runs (see Methods, Stage 2 and Supplementary Section 1.1) with the number of PG and DP molecules ranging from 2 to 7. The boxplot marks the mean (red dot) and the standard deviation (black error bars) of the cross-correlation coefficient (Bonomi et al., 2019) between the top 10% best-scoring models (based on their cross-correlation coefficient) and the cryo-tomogram in each of the runs. **(Inset)** The seven densities in the PKP layer of the tomogram (top view) of which four were full-length PKPs in our model (filled circles) and three were fixed, non-interacting PKP structured regions (empty circles).

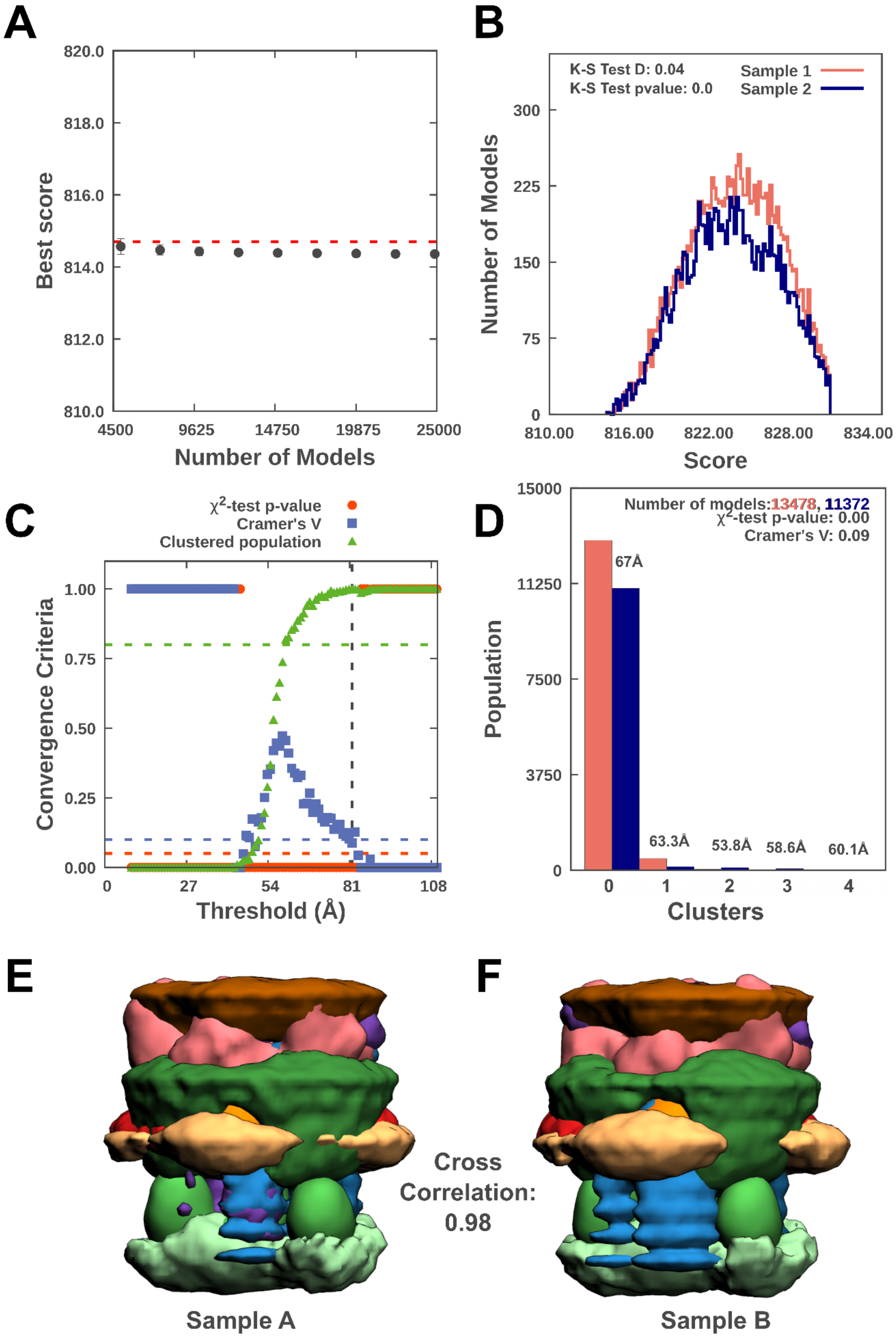
A
Best score
Number of Models
B
K-S Test D: 0.04
K-S Test pvalue: 0.0
Sample 1
Sample 2
Number of Models
Score
C
χ2-test p-value
Cramer's V
Clustered population
Convergence Criteria
Threshold (Å)
D
Number of models:13478, 11372
χ2-test p-value: 0.00
Cramer's V: 0.09
67Å
63.3Å
53.8Å
58.6Å
60.1Å
Population
Clusters
E
F
Cross Correlation: 0.98
Sample A
Sample B

Figure S2 Sampling Exhaustiveness protocol for Desmosomal ODP (Viswanath et al., 2017)) **A)** Test for the convergence of the model score for the 24016 good-scoring models. The scores do not continue to improve as more models are added independently. The error bar represents the standard deviations of the best scores, estimated by repeating sampling of models 10 times. The red dotted line indicates a lower bound reference on the total score. **B)** Testing the similarity of model score distributions between samples 1 (red) and 2 (blue). The difference in the distribution of scores is significant (Kolmogorov-Smirnov two-sample test p-value less than 0.05) but the magnitude of the difference is small (the Kolmogorov-Smirnov two-sample test statistic D is 0.04); thus, the two score distributions are effectively equal. **C)** Three criteria for determining the sampling precision (Y-axis), evaluated as a function of the RMSD clustering threshold (X-axis). First, the p-value is computed using the χ^2-test for homogeneity of proportions (red dots). Second, an effect size for the χ^2-test is quantified by the Cramer's V value (blue squares). Third, the population of models in sufficiently large clusters (containing at least 10 models from each sample) is shown as green triangles. The vertical dotted grey line indicates the RMSD clustering threshold at which three conditions are satisfied (p-value > 0.05 [dotted red line], Cramer's V < 0.10 [dotted blue line], and the population of clustered models > 0.80 [dotted green line]), thus defining the sampling precision of 82 Å. **D)** Populations of sample 1 and 2 models in the clusters obtained by threshold-based clustering using the RMSD threshold of 82 Å. Cluster precision is shown for each cluster **E-F)** Comparison of localization probability densities of models from sample A and sample B for the major cluster. The cross-correlation of the density maps (see Supplementary section 2.2) of the two samples is greater than 0.98.

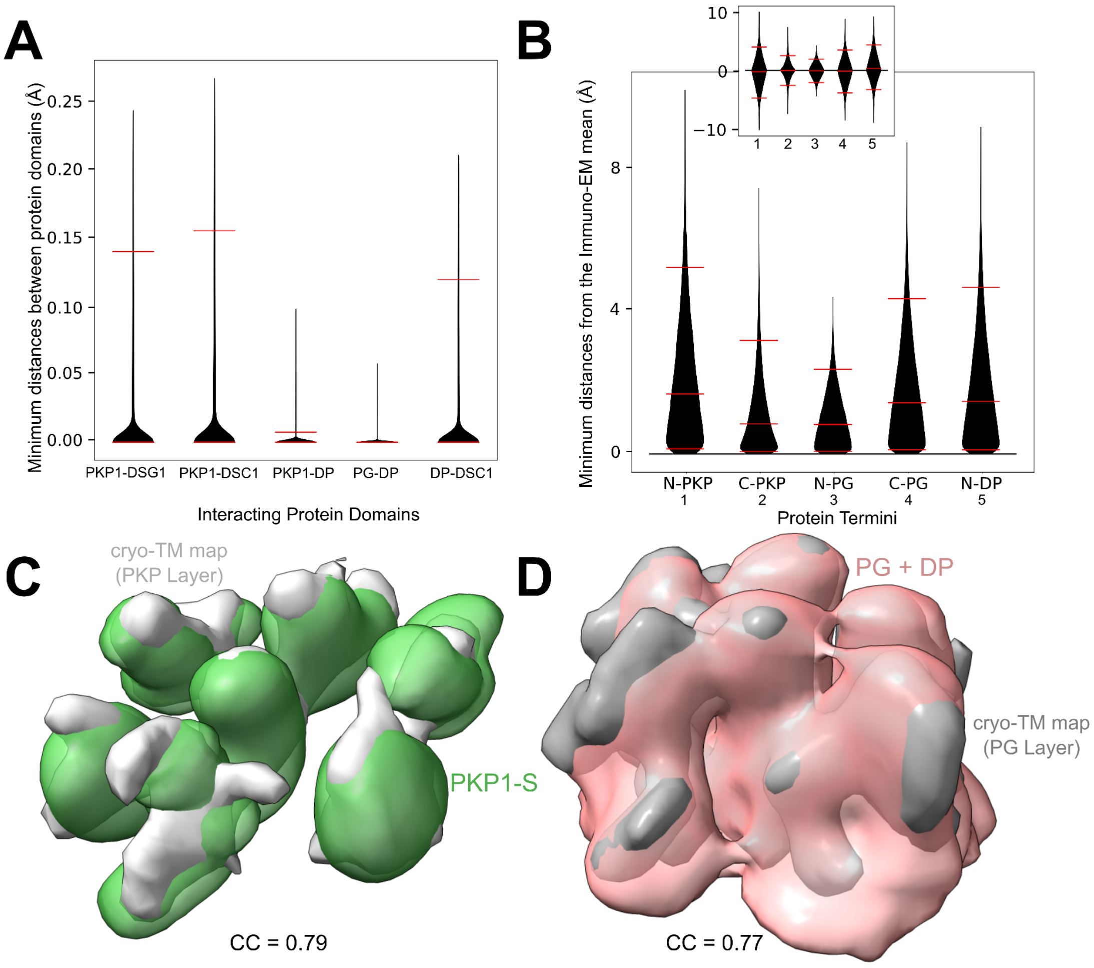

Figure S3 Fit to data used in modeling A) Fit to the data from biochemical experiments formulated as binding restraints (Methods). Each violin corresponds to the absolute closest distance between the two interacting domains across all protein copies for a model in the major cluster (Methods). Each distribution corresponds to a restraint in Table S2A. Red horizontal lines correspond to 5th, 50th and 95th percentile (in **A** and **B** both) after outlier removal. **B)** Fit to immuno-EM data (North et al., 1999). Each violin corresponds to the absolute difference between the experimental mean and the model-predicted distance from the membrane (Methods). The inset shows the same information without the absolute value (i.e. signed difference). **C-D)** Fit to the cryo-tomogram for the PKP Layer (**C**) and the PG Layer (**D**). Densities from the model (colored) are shown along with the segmented densities from the tomogram, EMD-1703 (Al-Amoudi et al., 2011)(Methods). The cross-correlation (CC) (Methods, Supplementary Section 2.2) is mentioned for each of the fits. PKP1-S density (including the non-interacting PKP1 molecules) and the PG + DP density are visualized at a ~10% threshold and the tomogram is visualized at the recommended threshold. See also Fig. 3, Table S2.

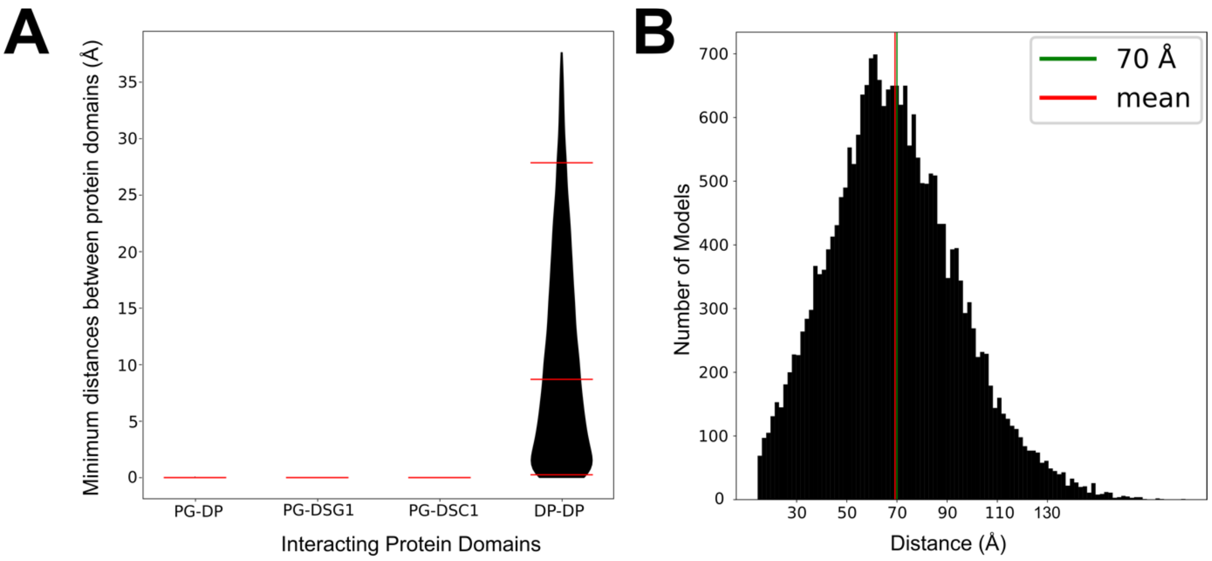

Figure S4 Fit to data not used in modeling A) Fit to the data from biochemical experiments not used for modeling (Methods). Each violin corresponds to the closest distance between the two interacting domains across all copies for a model in the major cluster (Methods). Each distribution corresponds to a row in Table S3. Red horizontal lines correspond to 5th, 50th and 95th percentile after outlier removal. See also Table S3. B) Fit to the cadherin spacing data from (Sikora et al., 2020). Histogram displays the minimum DSC1-DSG1 distance at the plasma membrane for each model. The data from Sikora *et*. al. is shown in green, and the mean of the distribution from integrative models is shown in red.

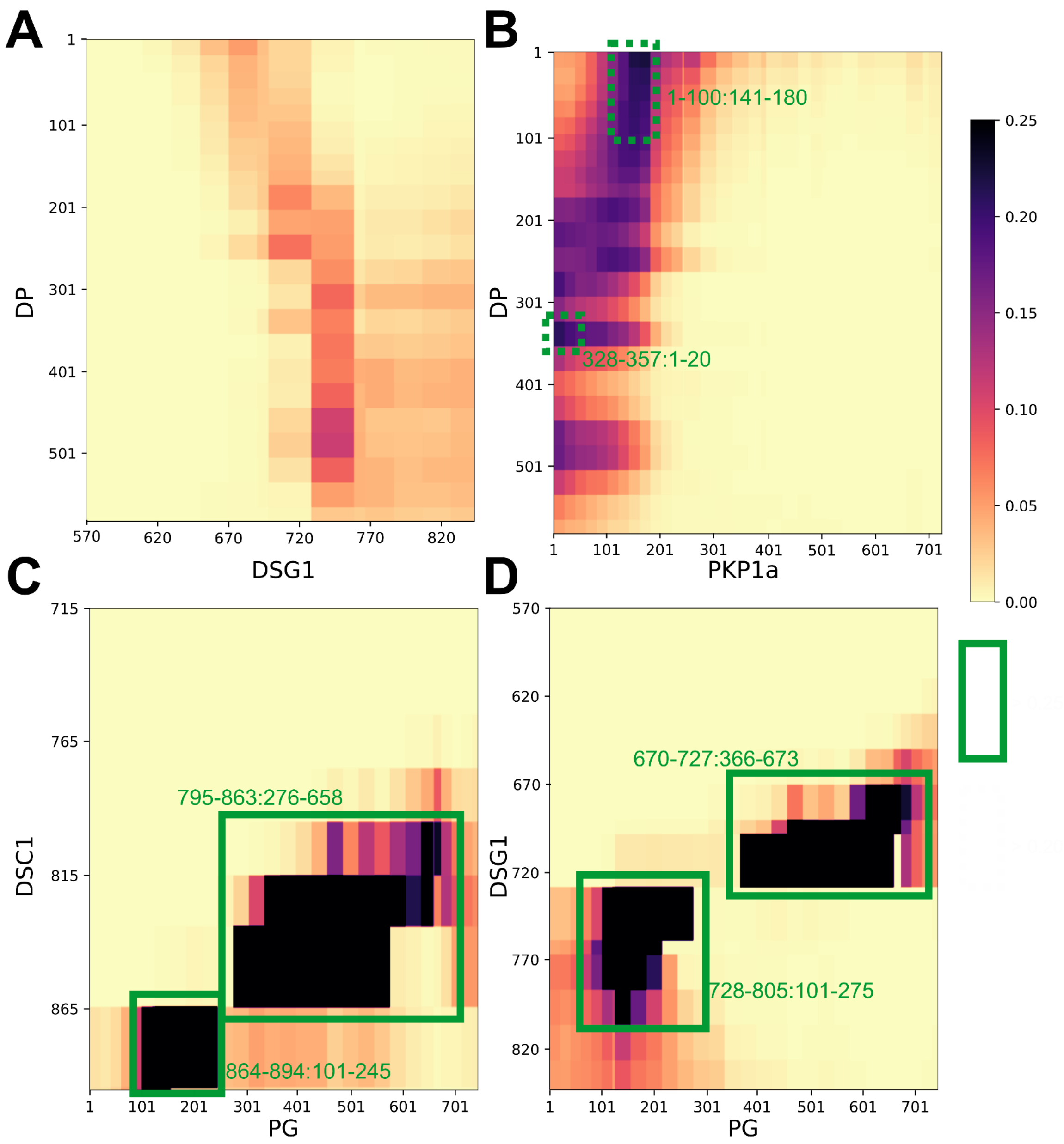

Figure S5 Additional contact maps Protein-protein contact maps for DP-DSG1 (**A**), DP-PKP1 (**B**), PG-DSC1 (**C**), and PG-DSG1 (**D**) pairs. Maps are colored by the proportion of the models in the major cluster where the corresponding two bead surfaces are within contact distance (10 Å). Rectangles with solid green (broken green) lines outline novel contacts present in >25% (>20%) of the models. See also Fig. 4 and Table S4.

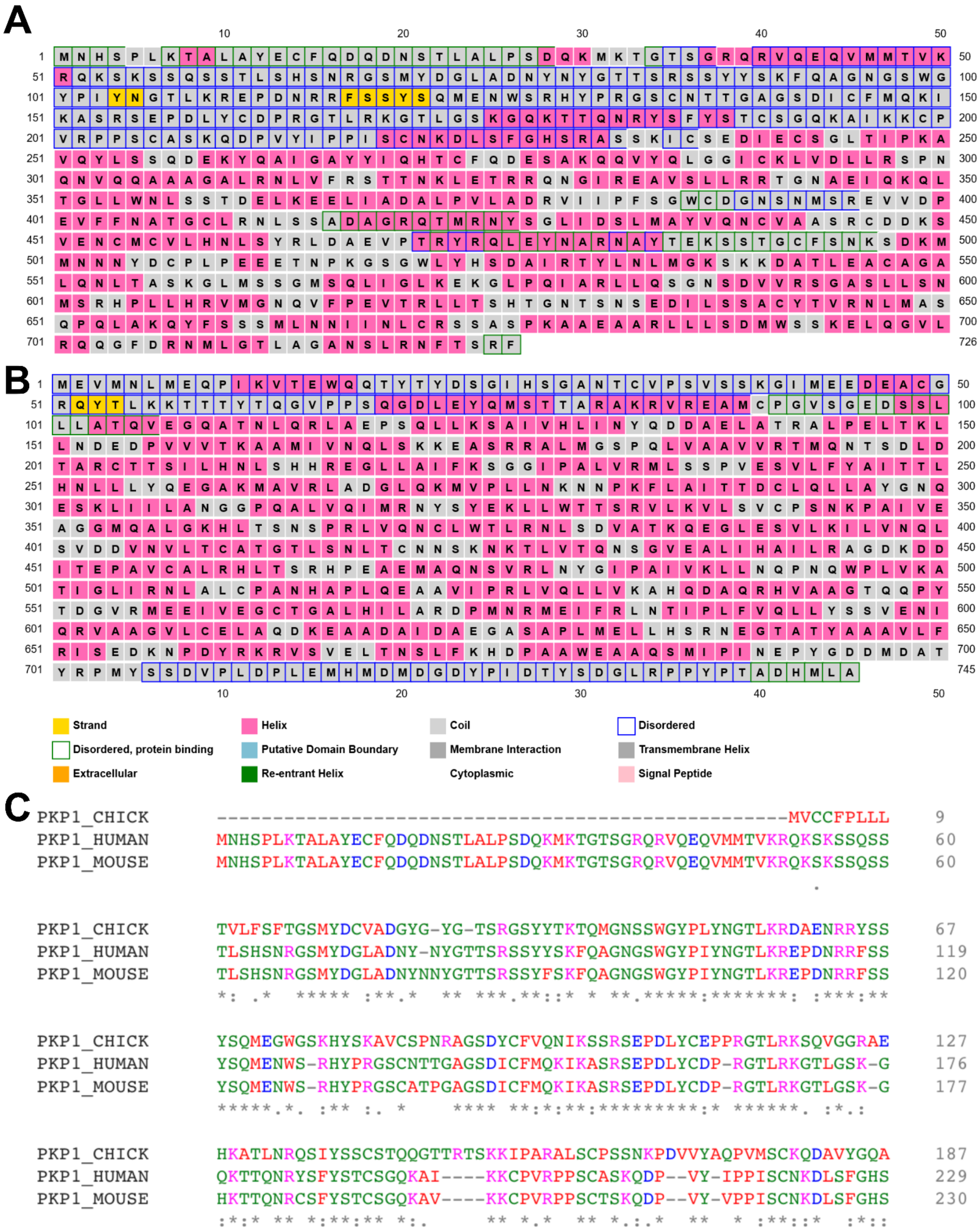

Figure S6 Prediction of disorder in PG and PKP1 and sequence conservation for PKP1
The PSIPRED (Buchan & Jones, 2019) output for PG (**A**) and PKP1 (**B**) are shown with the color scheme displayed below panel B. **C)** Sequence conservation output using Clustal Omega (Goujon et al., 2010; Sievers et al., 2011) for Chick, Human and Mouse PKP1$^{1-229}$. The colors represent different kinds of amino acids (acidic: blue, basic: magenta, hydrophobic/small: red,

Hydroxyl/sulfhydryl/amine/G: green). An asterisk (*) represents an exact residue match, a colon (:) represents a strongly similar match and a period (.) represents a weakly similar match.

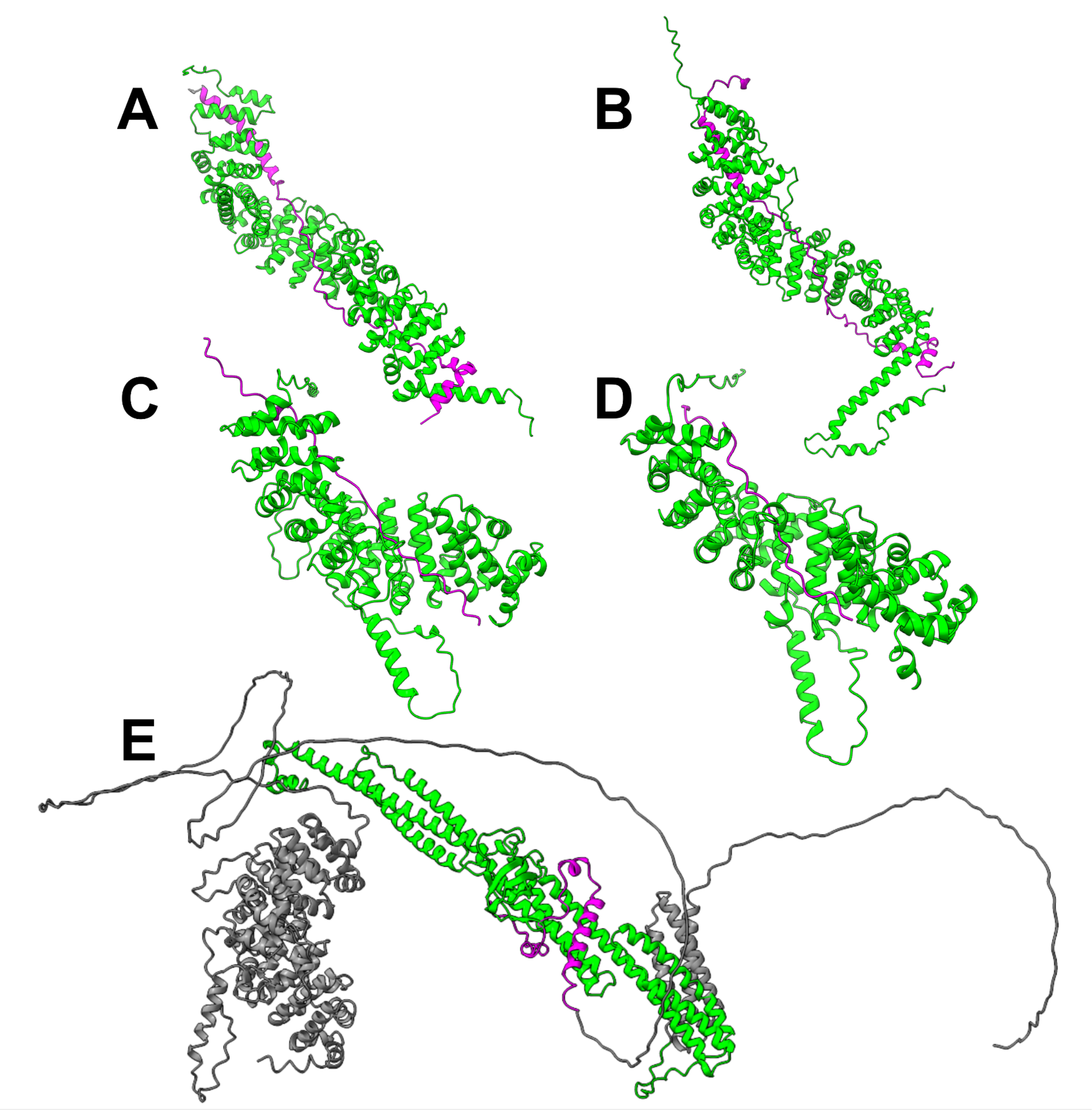

Figure S7 Alphafold2-Multimer predictions The best ranked model (based on PTM + IPTM score) is displayed for each protein pair. **A)** PG$^{107\text{-}697}$ (lime) and DSC1$^{812\text{-}894}$ (magenta) **B)** PG$^{75\text{-}699}$ (lime) and DSG1$^{680\text{-}768}$ (magenta) **C)** PKP1$^{229\text{-}747}$ (lime) and DSC1$^{722\text{-}759}$ (magenta) **D)** PKP1$^{230\text{-}744}$ (limer) and DSG1$^{712\text{-}737}$ (magenta) **E)** DP$^{160\text{-}584}$ (lime) and PKP1$^{1\text{-}60}$ (magenta). Rest of the residues of DP and PKP1 that are away from the interface are in gray. The PKP1 isoform used in **C, D, E** is PKP1b (in contrast to PKP1a used for integrative modeling in the paper) which has an extra 20-residue segment (PKP1b$^{412\text{-}432}$). This however does not affect any conclusions in the paper or the figure.

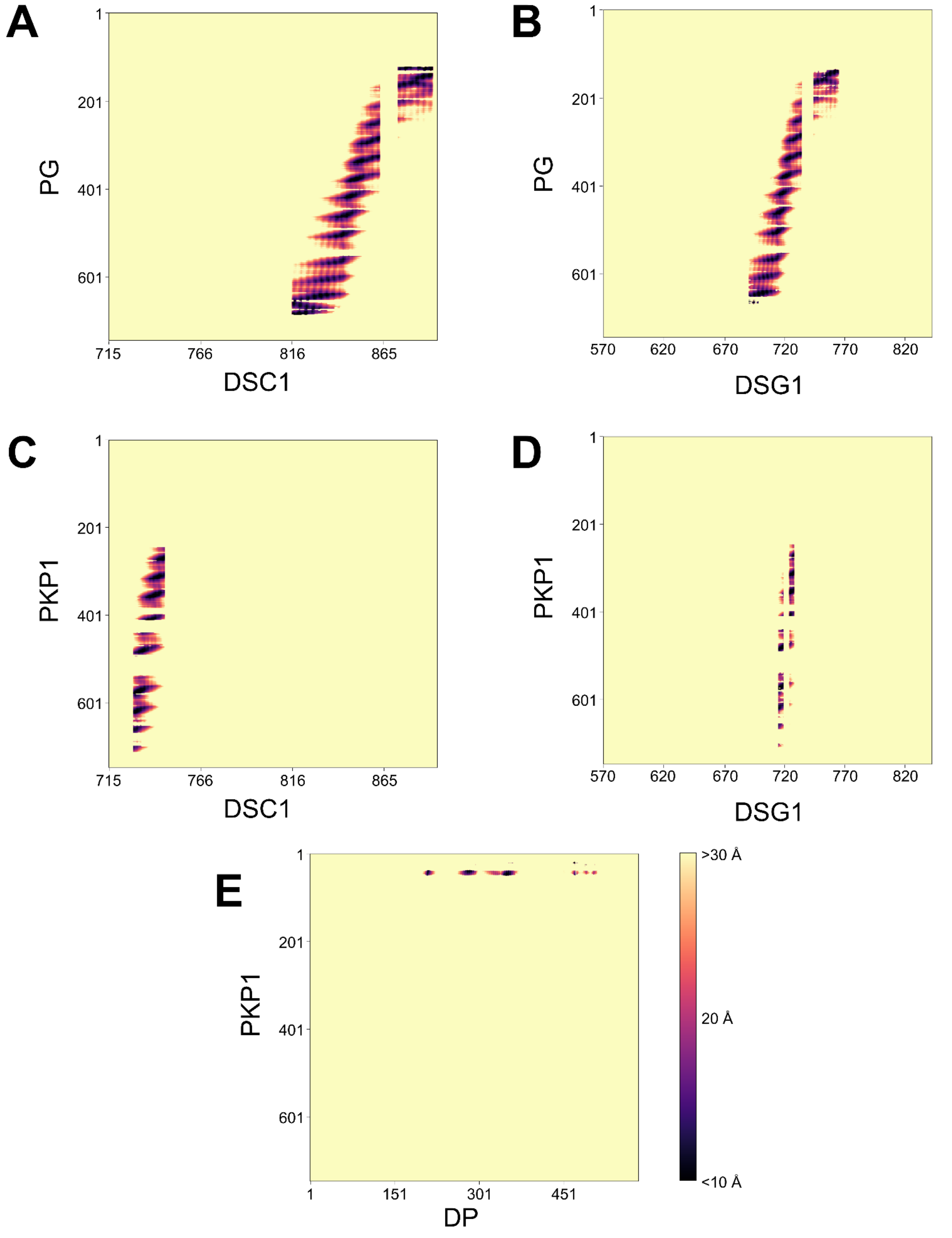

Figure S8 Contacts predicted by Alphafold2-Multimer Contact maps (Cα-Cα distance between residues) for PG-DSC1 (**A**), PG-DSG1 (**B**), PKP1-DSC1(**C**), PKP1-DSG1(**D**), and PKP1-DP (**E**) based on the best ranked AF2-Multimer model for each protein pair. The map is

colored by the distance between Cα atoms of a residue pair. Regions in contact distance (Cα-Cα distance <10Å) are colored black. Distances are shown only for residue pairs with reliable AF2 prediction (PAE <5 and pLDDT >70 for each residue in the pair). Residue pairs without reliable AF2 prediction are colored as >30Å irrespective of their actual Cα-Cα distance. The PKP1 isoform used in **C, D, E** is PKP1b as in Fig. S8.

Supplementary Tables

Protein	Residue Ranges	Structure: PDB (chain) or unknown	Uniprot ID
DP1	1-177	Unknown	P15924
	178-584	3R6N (A)	
PKP1a	1-243	Unknown	Q13835-2
	244-700	1XM9 (A)	
	701-726	Unknown	
PG	1-125	Unknown	P14923
	126-673	3IFQ (A)	
	674-745	Unknown	
DSG1a	570-697	Unknown	Q02413
	698-765	3IFQ (C)	
	766-842	Unknown	
DSC1a	715-833	Unknown	Q08554-1
	834-894	3IFQ (C)	

Table S1 Modeled protein domains The different domains of the modeled proteins are shown along with their residue ranges andstructure Regions of unknown structure were represented as flexible 20-residue beads, while regions of known structure were represented as rigid bodies consisting of 30-residue beads. The colors refer to domains without a known structure (red), domains which are homology modeled on a structure template of an isoform or homolog (yellow), and domains which have the structure in the PDB (green). Only the domains in the ODP were modeled; extracellular and transmembrane domains ($DSG1^{1-569}$, $DSC1^{1-714}$) or domains outside the ODP ($DP^{585-2871}$, $DSG1^{843-1049}$) were not modeled (Al-Amoudi et al., 2011; Garrod & Chidgey, 2008; Nilles et al., 1991).

Protein 1	Protein 2	Domain 1	Domain 2	Experiment	Reference	Data for Restraint Protein 1 residues: Protein 2 residues
PKP1a	DSG1	70-213	570-1049	Y2H	(Hatzfeld et al., 2000)	70-213:570-842
		1-286	568-1049	Y2H	(Kowalczyk et al., 1999)	
		1-726	499-1049	OA	(Smith & Fuchs, 1998)	
PKP1a	DSC1	1-726	715-894	OA	(Smith & Fuchs, 1998)	1-726:715-894
DP	DSC1	1-176	715-894	OA	(Smith & Fuchs, 1998)	1-176:715-894
PKP1a	DP	1-168	1-584	Y2H	(Hatzfeld et al., 2000)	1-168:1-584
		1-286	1-584	Y2H, co-IP, Loc	(Kowalczyk et al., 1999)	
		1-726	1-1014	OA	(Smith & Fuchs, 1998)	
		1-726	1-2871	Loc	(Bornslaeger et al., 2001)	
PG	DP	1-745	1-2871	OA	(Smith & Fuchs, 1998)	1-745:1-584
**The below data are on ODP isoforms that were not included in the main model.**						
PKP3a	DSC1	1-18 & 51-797	715-894	Y2H, co-IP, Loc	(Bonné et al., 2003)	(1-18 + 51-797):715-894
PKP3a	DSG1	1-18 & 51-293	519-715 Or 715-1k	Y2H, co-IP	(Bonné et al., 2003)	(1-18 + 51-293):570-842

PKP3a	DP	19-50	1-2871	Loc	(Bonné et al., 2003)	(1-18 + 51-293):1-584 & 19-50:1-63
		1-18 & 51-293	1-584	Y2H, co-IP, Loc	(Bonné et al., 2003)	
		19-50	1-63	Y2H	(Bonné et al., 2003)	
PKP3a	DSC3	1-18 & 51-797	712-897	Y2H, co-IP	(Bonné et al., 2003)	(1-18 + 51-797):712-897
PKP3a	DSG3	1-18 & 51-797	641-999	Y2H, co-IP	(Bonné et al., 2003)	(1-18 + 51-797):641-999

Table S2A Binding Restraints The protein-protein binding restraints are shown along with the experimental data they are based on. The restraint is formulated by including only the residues that are modeled in our ODP model. If multiple experiments provide data for a protein pair, the data from the experiment with the highest resolution is used. In Columns 3, 4 (and 5), green background (or text) represents the highest resolution information that was used to formulate the restraint and Gray represents other information that is at a lower resolution than the restraint. Experiment abbreviation are as follows: Y2H: Yeast 2 Hybrid, OA: Overlay Assay (in-vitro), Loc: Co-Localization assays, Co-IP: Co-Immunoprecipitation.

Protein Termini	Residues	Mean dist. (Å)	SE (Å)
DP-N	1-189	103	9.8
PG-N	1-106	229	4.5
PG-C	666-738	108	9
PKP1-N	1-285	158	11
PKP1-C	286-726	42	11
**The below data are on ODP isoforms that were not included in the main model.**			
PKP3-N	1-359	158	11
PKP3-C	360-797	42	11

Table S2B Immuno-EM Restraints The antibody binding domains for the different termini, mean distance of the termini from the plasma membrane, and the respective standard errors are shown (North et al., 1999). Domain names are in accordance with Fig. 1.

Protein 1	Protein 2	Domain 1	Domain 2	Experiment	Reference	Data for validation Protein 1 residues: Protein 2 residues
DP	DP	1-1014	1-176	OA	(Smith & Fuchs, 1998)	1-584:1-176
PG	DP	FULL	FULL	Y2H, co-IP	(Kowalczyk et al., 1997)	1-745:1-584
PG	DSG1	FULL	701-768	co-IP, Loc	(Troyanovsky, Troyanovsky, Eshkind, Krutovskikh, et al., 1994)	123-632:701-768
PG	DSG1	123-632	FULL	co-IP	(Wahl et al., 1996)	
PG	DSG1	FULL	663-958	ITC	(Choi et al., 2009)	
PG	DSC1	FULL	858-894	co-IP, Loc	(Troyanovsky, Troyanovsky, Eshkind, Leube, et al., 1994)	123-632: 858-894
PG	DSC1	123-632	FULL	co-IP	(Wahl et al., 1996)	
PG	DSC1	FULL	795-894	ITC	(Choi et al., 2009)	
DP	DSC1	FULL	728-740	co-IP, Loc	(Troyanovsky, Troyanovsky, Eshkind, Leube, et al., 1994)	1-584:728-740
PG	DSC1	FULL	728-740	co-IP, Loc	(Troyanovsky, Troyanovsky, Eshkind, Leube, et al., 1994)	123-632: 728-740

Table S3A Validation protein-protein binding data not used in modeling The protein-protein binding data and the corresponding references not used in modeling are shown. Data for validation was obtained using the same reasoning as in Table S2A. Experiment abbreviation are as follows: Y2H: Yeast 2 Hybrid, OA: Overlay Assay (in-vitro), Loc: Co-Localization assays, Co-IP: Co-Immunoprecipitation, ITC: Isothermal Calorimetry. Gray text represents the information that was not validated by the ensemble of models.

Restrained protein domain (s) or residues	Data for validation	Experiment	Reference
PG-N (PG^{1-106})	Distance of domain from membrane = 240 +/- 20 Å	dSTORM	(Stahley et al., 2016)
DP-N (DP^{1-189})	Distance of domain from membrane = 410 +/60 Å	dSTORM	(Stahley et al., 2016)
$DSG1^{570-589}$, $DSC1^{715-734}$	Cadherin spacing: Distance between DSG1 membrane-bound region and DSC1 membrane-bound region is 7 nm	cryoTM	(Sikora et al., 2020)
$DSG1^{571}$, $DSG1^{573}$	Residues are membrane-interacting	ABE	(Roberts et al., 2016)

Table S3B Other validation data not used in modeling Data from imaging and biochemistry and the corresponding references not used in modeling are shown. Experiment abbreviation are as follows: dSTORM: direct stochastic optical reconstruction microscopy, cryoTM: electron cryo-tomography, ABE: acyl biotin exchange assay. Gray text represents the information that was not validated by the ensemble of models.

Protein 1	Residues in protein 1	Protein 2	Residues in protein 2
DP	1-20	DSC1	795-833
DP	21-60	DSC1	815-833
DP	21-40	DSC1	795-814
DP	61-100	DSC1	815-833
DP	141-177	DSC1	864-893
DP	178-237	PG	306-335
DP	238-267	PG	276-335
DP	61-80	PG	366-395
DP	81-100	PG	336-395
DP	101-140, 161-177	PG	306-395
DP	141-160	PG	306-425
DP	178-207	PG	246-305, 336-425
DP	208-237	PG	216-305, 336-395
DP	238-267	PG	216-275, 336-455
DP	268-297	PG	246-275
DP	328-357	PG	216-335
DP	448-507	PG	81-120, 126-275
DP	508-537	PG	61-100
DP	1-60	PKP1	141-180
DP	61-100	PKP1	141-160
DP	328-357	PKP1	1-20
DSC1	795-814	PG	636-658

DSC1	815-833	PG	336-605, 636-658
DSC1*	834-863	PG	276-575
DSC1*	864-894	PG	101-245
DSC1*	894	PG	101-155
DSC1	795-814	PG	661-673
DSC1	815-833	PG	606-635
DSG1	670-689	PG	606-673
DSG1	690-697	PG	456-673
DSG1*	698-727	PG	366-658
DSG1*	728-757	PG	101-275
DSG1*	758-765	PG	101-215
DSG1	766-785	PG	101-185
DSG1	786-805	PG	126-155
DSG1	670-689	PG	674-693
DSG1*	698-727	PG	659-660
DSG1	766-785	PG	186-215
DSC1	775-794	PKP1	201-220
DSC1	795-814	PKP1	181-220
DSC1	795-814	PKP1	161-180
DSC1	815-833	PKP1	161-220
DSG1	650-669	PKP1	181-220
DSG1	670-689	PKP1	161-220
DSG1	630-649	PKP1	201-220
DSG1	650-669	PKP1	221-240
DSG1	670-689	PKP1	141-160

DSG1	690-697	PKP1	181-200

Table S4 Protein-Protein contacts in the ODP All the contacts (bead surface-to-surface distance of less than 10 Å) identified in at least 20% (yellow) to 25% (green) of the models in the ensemble are shown (Methods). Contacts consistent with sub-complexes of known structure are marked with an asterisk in column 1.

Protein Domain	Residues	Mutation	Reference	Disease
PG-N	19	T → I	(Den Haan et al., 2009)	Naxos disease
PG-S	265	R → H	(Erken et al., 2011)	Naxos disease
PG-S	301	E → G	(Marino et al., 2017)	Naxos disease
PG-C	680-745	WEAAQSMIPI → GGCPEHDSHQ + Δ690-745	(McKoy et al., 2000)	Naxos disease
DP-S	287	N → K	(Whittock et al., 2002)	Skin Fragility - Woolly Hair Syndrome
DP-S	356	T → K	(Pigors et al., 2015)	Carvajal syndrome
DP-S	564	T → I	(Boulé et al., 2012; Keller et al., 2012)	Carvajal syndrome
DP-S	583	L → P	(Keller et al., 2012)	Carvajal syndrome
PKP1-S	502	R → H	COSMIC	Cancer Mutation
PG-N	4	M → V	COSMIC	Cancer Mutation
DSG1	788	E → K	COSMIC	Cancer Mutation
DSC1	841	Y → F	COSMIC	Cancer Mutation

Table S5 Mutations The mutations of interest in the different protein domains are shown along with the pathology associated with the mutation (Fig. 5, Results). Domain names are in accordance with Fig. 1.

References

Al-Amoudi, A., Castaño-Diez, D., Devos, D. P., Russell, R. B., Johnson, G. T., & Frangakis, A. S. (2011). The three-dimensional molecular structure of the desmosomal plaque. *Proceedings of the National Academy of Sciences*, *108*(16), 6480–6485. https://doi.org/10.1073/pnas.1019469108

Alber, F., Dokudovskaya, S., Veenhoff, L. M., Zhang, W., Kipper, J., Devos, D., Suprapto, A., Karni-Schmidt, O., Williams, R., Chait, B. T., Rout, M. P., & Sali, A. (2007). Determining the architectures of macromolecular assemblies. *Nature*, *450*(7170), 683–694. https://doi.org/10.1038/nature06404

Bonné, S., Gilbert, B., Hatzfeld, M., Chen, X., Green, K. J., & Van Roy, F. (2003). Defining desmosomal plakophilin-3 interactions. *Journal of Cell Biology*, *161*(2), 403–416. https://doi.org/10.1083/jcb.200303036

Bonomi, M., Hanot, S., Greenberg, C. H., Sali, A., Nilges, M., Vendruscolo, M., & Pellarin, R. (2019). Bayesian Weighing of Electron Cryo-Microscopy Data for Integrative Structural Modeling. *Structure*, *27*(1), 175-188.e6. https://doi.org/10.1016/j.str.2018.09.011

Bornslaeger, E. A., Godsel, L. M., Corcoran, C. M., Park, J. K., Hatzfeld, M., Kowalczyk, A. P., & Green, K. J. (2001). Plakophilin 1 interferes with plakoglobin binding to desmoplakin, yet together with plakoglobin promotes clustering of desmosomal plaque complexes at cell-cell borders. *Journal of Cell Science*, *114*(4), 727–738. https://doi.org/10.1242/jcs.114.4.727

Boulé, S., Fressart, V., Laux, D., Mallet, A., Simon, F., De Groote, P., Bonnet, D., Klug, D., & Charron, P. (2012). Expanding the phenotype associated with a desmoplakin dominant mutation: Carvajal/Naxos syndrome associated with leukonychia and oligodontia.

International Journal of Cardiology, *161*(1), 50–52. https://doi.org/10.1016/j.ijcard.2012.06.068

Buchan, D. W. A., & Jones, D. T. (2019). The PSIPRED Protein Analysis Workbench: 20 years on. *Nucleic Acids Research*, *47*(W1), W402–W407. https://doi.org/10.1093/nar/gkz297

Chodera, J. D. (2016). A Simple Method for Automated Equilibration Detection in Molecular Simulations. *Journal of Chemical Theory and Computation*, *12*(4), 1799–1805. https://doi.org/10.1021/acs.jctc.5b00784

Choi, H.-J., Gross, J. C., Pokutta, S., & Weis, W. I. (2009). Interactions of Plakoglobin and β-Catenin with Desmosomal Cadherins. *Journal of Biological Chemistry*, *284*(46), 31776–31788. https://doi.org/10.1074/jbc.M109.047928

Den Haan, A. D., Tan, B. Y., Zikusoka, M. N., Lladó, L. I., Jain, R., Daly, A., Tichnell, C., James, C., Amat-Alarcon, N., Abraham, T., Russell, S. D., Bluemke, D. A., Calkins, H., Dalal, D., & Judge, D. P. (2009). Comprehensive Desmosome Mutation Analysis in North Americans With Arrhythmogenic Right Ventricular Dysplasia/Cardiomyopathy. *Circulation: Cardiovascular Genetics*, *2*(5), 428–435. https://doi.org/10.1161/CIRCGENETICS.109.858217

Erken, H., Yariz, K. O., Duman, D., Kaya, C. T., Sayin, T., Heper, A. O., & Tekin, M. (2011). Cardiomyopathy with alopecia and palmoplantar keratoderma (CAPK) is caused by a *JUP* mutation. *British Journal of Dermatology*, *165*(4), 917–921. https://doi.org/10.1111/j.1365-2133.2011.10455.x

Garrod, D., & Chidgey, M. (2008). Desmosome structure, composition and function. *Biochimica et Biophysica Acta (BBA) - Biomembranes*, *1778*(3), 572–587. https://doi.org/10.1016/j.bbamem.2007.07.014

Goujon, M., McWilliam, H., Li, W., Valentin, F., Squizzato, S., Paern, J., & Lopez, R. (2010). A new bioinformatics analysis tools framework at EMBL-EBI. *Nucleic Acids Research*, *38*(Web Server), W695–W699. https://doi.org/10.1093/nar/gkq313

Hatzfeld, M., Haffner, C., Schulze, K., & Vinzens, U. (2000). The Function of Plakophilin 1 in Desmosome Assembly and Actin Filament Organization. *Journal of Cell Biology*, *149*(1), 209–222. https://doi.org/10.1083/jcb.149.1.209

Keller, D., Stepowski, D., Balmer, C., Simon, F., Guenthard, J., Bauer, F., Itin, P., David, N., Drouin-Garraud, V., & Fressart, V. (2012). De novo heterozygous desmoplakin mutations leading to Naxos-Carvajal disease. *Swiss Medical Weekly*. https://doi.org/10.4414/smw.2012.13670

Kohn, J. E., Millett, I. S., Jacob, J., Zagrovic, B., Dillon, T. M., Cingel, N., Dothager, R. S., Seifert, S., Thiyagarajan, P., Sosnick, T. R., Hasan, M. Z., Pande, V. S., Ruczinski, I., Doniach, S., & Plaxco, K. W. (2004). Random-coil behavior and the dimensions of chemically unfolded proteins. *Proceedings of the National Academy of Sciences*, *101*(34), 12491–12496. https://doi.org/10.1073/pnas.0403643101

Kowalczyk, A. P., Bornslaeger, E. A., Borgwardt, J. E., Palka, H. L., Dhaliwal, A. S., Corcoran, C. M., Denning, M. F., & Green, K. J. (1997). The Amino-terminal Domain of Desmoplakin Binds to Plakoglobin and Clusters Desmosomal Cadherin–Plakoglobin Complexes. *Journal of Cell Biology*, *139*(3), 773–784. https://doi.org/10.1083/jcb.139.3.773

Kowalczyk, A. P., Hatzfeld, M., Bornslaeger, E. A., Kopp, D. S., Borgwardt, J. E., Corcoran, C. M., Settler, A., & Green, K. J. (1999). The Head Domain of Plakophilin-1 Binds to Desmoplakin and Enhances Its Recruitment to Desmosomes. *Journal of Biological Chemistry*, *274*(26), 18145–18148. https://doi.org/10.1074/jbc.274.26.18145

Marino, T. C., Maranda, B., Leblanc, J., Pratte, A., Barabas, M., Dupéré, A., & Lévesque, S. (2017). Novel founder mutation in French-Canadian families with Naxos disease: Letter to the Editor. *Clinical Genetics*, *92*(4), 451–453. https://doi.org/10.1111/cge.12971

McKoy, G., Protonotarios, N., Crosby, A., Tsatsopoulou, A., Anastasakis, A., Coonar, A., Norman, M., Baboonian, C., Jeffery, S., & McKenna, W. J. (2000). Identification of a deletion in plakoglobin in arrhythmogenic right ventricular cardiomyopathy with

palmoplantar keratoderma and woolly hair (Naxos disease). *The Lancet*, *355*(9221), 2119–2124. https://doi.org/10.1016/S0140-6736(00)02379-5

Nilles, L. A., Parry, D. A. D., Powers, E. E., Angst, B. D., Wagner, R. M., & Green, K. J. (1991). Structural analysis and expression of human desmoglein: A cadherin-like component of the desmosome. *Journal of Cell Science*, *99*(4), 809–821. https://doi.org/10.1242/jcs.99.4.809

North, A. J., Bardsley, W. G., Hyam, J., Bornslaeger, E. A., Cordingley, H. C., Trinnaman, B., Hatzfeld, M., Green, K. J., Magee, A. I., & Garrod, D. R. (1999). Molecular map of the desmosomal plaque. *Journal of Cell Science*, *112*(23), 4325–4336. https://doi.org/10.1242/jcs.112.23.4325

Pigors, M., Schwieger-Briel, A., Cosgarea, R., Diaconeasa, A., Bruckner-Tuderman, L., Fleck, T., & Has, C. (2015). Desmoplakin Mutations with Palmoplantar Keratoderma, Woolly Hair and Cardiomyopathy. *Acta Dermato Venereologica*, *95*(3), 337–340. https://doi.org/10.2340/00015555-1974

Roberts, B. J., Svoboda, R. A., Overmiller, A. M., Lewis, J. D., Kowalczyk, A. P., Mahoney, M. G., Johnson, K. R., & Wahl, J. K. (2016). Palmitoylation of Desmoglein 2 Is a Regulator of Assembly Dynamics and Protein Turnover. *Journal of Biological Chemistry*, *291*(48), 24857–24865. https://doi.org/10.1074/jbc.M116.739458

Saltzberg, D. J., Viswanath, S., Echeverria, I., Chemmama, I. E., Webb, B., & Sali, A. (2021). Using Integrative Modeling Platform to compute, validate, and archive a model of a protein complex structure. *Protein Science*, *30*(1), 250–261. https://doi.org/10.1002/pro.3995

Sievers, F., Wilm, A., Dineen, D., Gibson, T. J., Karplus, K., Li, W., Lopez, R., McWilliam, H., Remmert, M., Söding, J., Thompson, J. D., & Higgins, D. G. (2011). Fast, scalable generation of high-quality protein multiple sequence alignments using Clustal Omega. *Molecular Systems Biology*, *7*(1), 539. https://doi.org/10.1038/msb.2011.75

Sikora, M., Ermel, U. H., Seybold, A., Kunz, M., Calloni, G., Reitz, J., Vabulas, R. M., Hummer, G., & Frangakis, A. S. (2020). Desmosome architecture derived from molecular dynamics simulations and cryo-electron tomography. *Proceedings of the National Academy of Sciences*, *117*(44), 27132–27140. https://doi.org/10.1073/pnas.2004563117

Smith, E. A., & Fuchs, E. (1998). Defining the Interactions Between Intermediate Filaments and Desmosomes. *Journal of Cell Biology*, *141*(5), 1229–1241. https://doi.org/10.1083/jcb.141.5.1229

Stahley, S. N., Bartle, E. I., Atkinson, C. E., Kowalczyk, A. P., & Mattheyses, A. L. (2016). Molecular organization of the desmosome as revealed by direct stochastic optical reconstruction microscopy. *Journal of Cell Science*, jcs.185785. https://doi.org/10.1242/jcs.185785

Teraoka, I. (2002). *Polymer solutions: An introduction to physical properties*. 2 John Wiley & Sons, Inc.

Troyanovsky, S. M., Troyanovsky, R. B., Eshkind, L. G., Krutovskikh, V. A., Leube, R. E., & Franke, W. W. (1994). Identification of the plakoglobin-binding domain in desmoglein and its role in plaque assembly and intermediate filament anchorage. *Journal of Cell Biology*, *127*(1), 151–160. https://doi.org/10.1083/jcb.127.1.151

Troyanovsky, S. M., Troyanovsky, R. B., Eshkind, L. G., Leube, R. E., & Franke, W. W. (1994). Identification of amino acid sequence motifs in desmocollin, a desmosomal glycoprotein, that are required for plakoglobin binding and plaque formation. *Proceedings of the National Academy of Sciences*, *91*(23), 10790–10794. https://doi.org/10.1073/pnas.91.23.10790

Virtanen, P., Gommers, R., Oliphant, T. E., Haberland, M., Reddy, T., Cournapeau, D., Burovski, E., Peterson, P., Weckesser, W., Bright, J., Van Der Walt, S. J., Brett, M., Wilson, J., Millman, K. J., Mayorov, N., Nelson, A. R. J., Jones, E., Kern, R., Larson, E., … Vázquez-Baeza, Y. (2020). SciPy 1.0: Fundamental algorithms for scientific

computing in Python. *Nature Methods*, *17*(3), 261–272. https://doi.org/10.1038/s41592-019-0686-2

Viswanath, S., Chemmama, I. E., Cimermancic, P., & Sali, A. (2017). Assessing Exhaustiveness of Stochastic Sampling for Integrative Modeling of Macromolecular Structures. *Biophysical Journal*, *113*(11), 2344–2353. https://doi.org/10.1016/j.bpj.2017.10.005

Wahl, J. K., Sacco, P. A., McGranahan-Sadler, T. M., Sauppe, L. M., Wheelock, M. J., & Johnson, K. R. (1996). Plakoglobin domains that define its association with the desmosomal cadherins and the classical cadherins: Identification of unique and shared domains. *Journal of Cell Science*, *109*(5), 1143–1154. https://doi.org/10.1242/jcs.109.5.1143

Whittock, N. V., Wan, H., Eady, R. A. J., Morley, S. M., Garzon, M. C., Kristal, L., Hyde, P., Irwin McLean, W. H., Pulkkinen, L., Uitto, J., Christiano, A. M., & McGrath, J. A. (2002). Compound Heterozygosity for Non-Sense and Mis-Sense Mutations in Desmoplakin Underlies Skin Fragility/Woolly Hair Syndrome. *Journal of Investigative Dermatology*, *118*(2), 232–238. https://doi.org/10.1046/j.0022-202x.2001.01664.x
